# Strategies to Increase Equitable Access to Penicillin Allergy Delabeling Interactions: A Qualitative Study

**DOI:** 10.1001/jamanetworkopen.2026.33825

**Published:** 2026-09-15

**Authors:** Alysse Wurcel, Danielle Crabtree, Olabimpe Asupoto, Madelyn Eippert, Yvane Ngassa, Rachel Tam, Ivana Kohut, Kap Sum Foong, Valerie Stone, Denise Daudelin, Stephen Bartels, Kimberly G. Blumenthal

**Affiliations:** 1 Section of General Internal Medicine, Boston Medical Center, Boston, Massachusetts; 2 Chobanian and Avedisian School of Medicine, Boston University, Boston, Massachusetts; 3 Section of Infectious Diseases, Boston Medical Center, Boston, Massachusetts; 4 Division of Allergy and Clinical Immunology, Massachusetts General Hospital, Boston; 5 Division of Geographic Medicine and Infectious Diseases, Tufts Medical Center, Boston, Massachusetts; 6 Division of Pulmonary Allergy and Sleep, Mayo Clinic, Jacksonville, Florida; 7 Division of General Internal Medicine & Primary Care, Brigham and Women’s Hospital, Boston, Massachusetts; 8 Department of Medicine, Harvard Medical School, Boston, Massachusetts; 9 Tufts Clinical and Translational Science Institute, Tufts University, Boston, Massachusetts; 10 Institute for Clinical Research and Health Policy Studies, Tufts Medical Center, Boston, Massachusetts; 11 The Mongan Institute, Massachusetts General Hospital, Boston, Massachusetts

## Abstract

**Importance:**

Patients with unconfirmed and potentially false penicillin allergy labels receive unnecessarily broad-spectrum antibiotics that can lead to worse health outcomes and antimicrobial resistance. While ambulatory care is a favorable setting to engage in penicillin allergy delabeling, the process of removing an incorrect penicillin allergy label has not been widely implemented.

**Objective:**

To contextualize barriers to and facilitators of penicillin allergy delabeling from a diverse range of perspectives and inform actionable strategies.

**Design, Setting, and Participants:**

This qualitative study used in-depth interviews and focus groups with patients who had a penicillin allergy label, clinicians, and other stakeholders from primary care clinics in 3 hospital networks in Boston, Massachusetts. Participants were recruited from March 1 through September 30, 2024.

**Main Outcome and Measures:**

The primary outcome was participant-perceived factors influencing engagement in discussions about penicillin allergy delabeling. The analytic approach was informed by the updated Consolidated Framework for Implementation Research (CFIR), with a mixed inductive-deductive analysis that incorporated the CFIR Expert Recommendations for Implementing Change (ERIC) implementation strategy matching tool to map interview data to implementation strategies.

**Results:**

The study included 119 participants (84 [71%] female), of whom 69 (58%) were patients and 50 (42%) were clinicians and stakeholders; 87 (73%) participated in interviews and 32 (27%) participated in focus groups. Six barriers to penicillin allergy delabeling emerged: (1) clinician avoidance of conversations about penicillin allergy labels, (2) patients not being asked about their penicillin allergy labels, (3) language barriers, (4) time constraints, (5) perceived elevated risk of removing a penicillin allergy, and (6) limited access to allergists. These barriers aligned with 5 ERIC strategies: (1) create new ways of delivering care, (2) identify champions, (3) use data experts, (4) alter incentive structures, and (5) conduct educational meetings.

**Conclusions and Relevance:**

This qualitative study guided by implementation science identified strategies to overcome barriers to penicillin allergy delabeling. These strategies may inform the next steps for implementation science research to improve access to penicillin allergy testing and delabeling.

## Key Points

**Question:**

What are the barriers to and facilitators of penicillin allergy delabeling in primary care settings?

**Findings:**

In this qualitative study of 119 patients, clinicians, and stakeholders, 6 barriers emerged: clinicians avoiding conversations about penicillin allergy labels, patients not being asked about penicillin allergy labels, language barriers, time constraints, perceived elevated risk of removing penicillin allergy labels, and limited access to allergists. These barriers mapped to 5 implementation strategies: creating new ways of delivering care, identifying champions, using data experts, altering incentive structures, and conducting educational meetings.

**Meaning:**

The findings offer several strategies that may improve access to penicillin allergy delabeling.

## Teaser

This qualitative study explores perspectives of patients with a penicillin allergy label, clinicians, and other stakeholders about penicillin allergy evaluation and potential delabeling in primary care settings.

## Introduction

Interventions aimed at decreasing unnecessary antibiotic use are crucial to combat the increasing threat of antibiotic resistance.^1^ As penicillin is well tolerated and an effective first line-treatment for many infections, people who report penicillin allergy often receive second-line antibiotics that may have more associated adverse effects.^2^ More than 20 million antibiotic decisions per year may be affected by an unverified and potentially false penicillin allergy.^2^ As over 95% of people who are labeled with a penicillin allergy are not truly allergic, there is robust support for penicillin allergy evaluations and the delabeling of mislabeled penicillin allergies.^3^ Penicillin allergy evaluation is recommended by several professional societies and public health agencies, including the US Centers for Disease Control and Prevention.^4,5^

The process of penicillin allergy evaluation begins with asking specific questions about the allergy to determine whether the allergy label can be removed based on history alone or if the patient would benefit from penicillin allergy diagnostic testing. Adverse effects not consistent with allergy, such as gastrointestinal symptoms, headache, or fatigue, should prompt delabeling by clinicians without need for referral to an allergist.^2^ High-risk allergy history, such as anaphylaxis, can result in referrals to allergists for validated penicillin allergy testing. Low-risk allergy symptoms, such as benign rashes, may elicit a delabeling procedure by which amoxicillin is administered under observation.^3^

Ideally, all people with a penicillin allergy would be evaluated by an allergist, but the lack of an adequate specialized workforce makes this unfeasible,^6^ especially in rural areas.^7^ Outpatient appointments with primary care are an optimal time to initiate the process of penicillin allergy delabeling, either directly or through appropriate discussion and referral for testing. Surveys of clinicians and people with penicillin allergy have identified barriers to implementing primary care–based penicillin allergy assessments, including fear of allergic reaction and perceived equal efficacy and safety of alternative antibiotics.^8,9^ Qualitative work to contextualize survey results has focused on engagement of only 1 stakeholder group (eg, patients or clinicians),^10,11^ preventing direct comparison of perspectives.

The current body of qualitative work has emerged from largely international health settings and mostly among White, English-speaking people.^12,13^ The predominance of people identifying as White in research about penicillin allergy is not surprising, as several studies have shown a positive association between penicillin allergy labels and White race.^13,14^ However, it is worth noting that the association is based on differential treatment patterns based on color of skin and not biology^15,16,17^ and there is robust literature demonstrating that antimicrobial resistance and inappropriate antibiotic use disproportionately affect and are affected by racial, ethnic, and gender-based health disparities.^18,19^ Health care systems vary among countries, and the lessons previously gleaned internationally may not be generalizable to the US, a country without a national health care system. Previous research by our team found an inverse association between social vulnerability and penicillin allergy delabeling in the greater Boston, Massachusetts, area.^20^ To our knowledge, the qualitative analyses published to date did not examine race, ethnicity, language, financial resources, or insurance—important contextual information to support interventions developed to increase access to penicillin allergy delabeling.

Although penicillin allergy delabeling is an evidence-based intervention, the process of delabeling requires shared decision-making. Implementation science methods can be harnessed to better understand the facilitators and barriers impacting clinician and patient engagement in shared decision-making to pursue penicillin allergy delabeling. We analyzed the perspectives of multiple stakeholders, including patients, clinicians, and other key informants with topical expertise, on factors believed to impact engagement in discussions about penicillin allergy delabeling as an intervention and strategies that can facilitate increased access to penicillin allergy evaluation in primary care clinics.

## Methods

### Frameworks

In this qualitative study, we used a hybrid deductive-inductive approach, also known as a framework method.^21^ Our theory-driven, deductive analytic approach was guided by an equity-focused adaptation of the updated Consolidated Framework for Implementation Research (CFIR), enabling systematic examination of multilevel implementation determinants. The CFIR is organized into 5 domains: (1) the intervention, (2) the outer setting, (3) the inner setting, (4) the individual, and (5) the process (eFigure in Supplement 1).^22^ We augmented the CFIR through use of a framework that guides research and interventions addressing health disparities.^23,24^ The framework guided the initial interview and focus group guide development, and coinvestigator review added questions to also support inductive analysis. The interview guides for patients, clinicians, and other stakeholders are included in eAppendixes 1-3 in Supplement 1. We acknowledge that the updated CFIR advises against collection of information from patients about implementation practices.^25^ The interview guide that focused on patients probed interest in the innovation to support methods of developing future implementation strategies. We used the Expert Recommendations for Implementing Change (ERIC) tool to support mapping themes about barriers and facilitators to strategies for change.^26^ The study was approved by the institutional review boards of Massachusetts Brigham General and Tufts University; participants provided verbal informed consent. We followed the Consolidated Criteria for Reporting Qualitative Research (COREQ) reporting guideline.

### Setting

With broad engagement across 6 primary care clinics from 3 hospital networks, we intentionally recruited patients, clinicians, and stakeholders from diverse backgrounds to better understand their views on penicillin allergy and develop strategies to improve equitable access to penicillin allergy evaluation and delabeling. We recruited patients, clinicians, and other key stakeholders with expertise about penicillin allergy.^27^ Race and ethnicity were collected to contextualize participant experiences and perceived barriers to penicillin allergy delabeling and were ascertained by self-report. Categories were Asian, non-Hispanic Black (hereafter, Black), Hispanic (any race), non-Hispanic White (hereafter, White), and multiracial or multiethnic. We aimed to enroll more than 50% of patients who identified their race and ethnicity as other than White. The included hospitals provide care to people in the greater Boston area, which encompasses a wide range of racial and ethnic groups (8% Black or African American, 12% Hispanic or Latino, 9% biracial, and 7% other race and ethnicity).^28^ Brigham and Women’s Hospital (BWH) provides care to a large portion of the Black population in Boston, especially individuals living in the neighborhoods of Dorchester, Mattapan, and Roxbury^29^ and including people from Haiti, the Dominican Republic, and Puerto Rico. Massachusetts General Hospital (MGH) cares for a large Spanish-speaking population, particularly at the Chelsea Community Health Center, where 50% of patients identify as Latino and Spanish is the preferred language for 30% of patients.^30^ Tufts Medical Center (TMC), based in Boston’s Chinatown, provides care to a predominantly Asian and Cantonese-speaking community.^31^

### Eligibility and Recruitment

We defined eligible patients as people who (1) received primary care at BWH, MGH, TMC, or their affiliated networks and (2) had a penicillin allergy label at any time from January 1, 2015, to December 31, 2022. We defined eligible clinicians as physicians, nurse practitioners, physician assistants, nurses (registered and licensed practical), and other health care staff in a predominantly patient-facing role and practicing in an outpatient primary care setting. Other stakeholders included people who worked in public health, insurance, a medical-related industry, information technology, and/or academic research. Patient participants were recruited through on-site clinic recruitment (eg, flyers, information tables) and physician referrals. Clinicians were recruited through direct solicitation supported by hospital, clinic, and nursing leadership. Clinicians were contacted through email, with a list of the employees’ emails obtained from the leadership teams of primary care clinics. Other stakeholders were identified through professional and community networks. Participants were remunerated with a $50 gift card and travel reimbursement.

### Data Collection

Recruitment occurred from March through September 2024. Using a research team of 7 people (included A.W., D.C., O.A., M.E., I.K., and K.S.F.), we conducted semistructured, in-depth qualitative interviews (approximately 60 minutes) and focus groups (approximately 90 minutes). Verbal participant consent was obtained to audio-record the interviews; however, participants could opt out of recording, in which case, the research staff took written notes instead. All in-depth interviews with patients were conducted in person. In-depth interviews with clinicians or other stakeholders were conducted in person or virtually over a videoconferencing platform. Interview guides were translated into Spanish, Mandarin, and Cantonese.

### Data Analyses

A professional transcription agency transcribed digital recordings verbatim. A mixed inductive-deductive framework guided the analysis. We developed the initial codebook based on the interview guides and supplemented the codes with emergent codes from the transcripts.^32^ We used Dedoose (SocioCultural Research Consultants, LLC), a qualitative management software program, to support analysis.^33^ To improve reliability and to ensure adequate intercoder agreement, 3 team members (D.C., O.A., Y.N.) independently coded transcripts, compared coding patterns, and refined the codebook until reaching consensus.^34^ We compiled quotations illustrating core emergent subthemes and themes. We compared the final codes and grouped them into themes and subthemes, which were then discussed, refined, and named for the final analysis. This thematic analysis identified barriers to penicillin allergy evaluation. We then mapped the barriers to ERIC implementation strategies and selected exemplar quotes to support each strategy. The final codebook is included as eAppendix 4 in Supplement 1.

## Results

A total of 176 individuals were approached for the study, and we enrolled 119 participants (69 patients [58%] and 50 clinicians or stakeholders [42%]); 84 (71%) were female and 35 (29%) were male. Nineteen participants (16%) were Asian, 14 (12%) were Black, 18 (15%) were Hispanic, 64 (54%) were White, and 4 (3%) were multiracial or multiethnic. A total of 87 participants (51 patients [59%], 30 clinicians [34%], and 6 stakeholders [7%]) participated in individual interviews, and 32 (18 patients [56%] and 14 clinicians [44%]) participated in focus groups. Four people (3%), all patients, participated in both an in-depth interview and a focus group (Table 1). Forty-six participants (39%) were from BWH, 28 (24%) from MGH, and 40 (34%) from TMC; 5 people (4%) were not affiliated with any of the 3 hospital systems. In the clinician group, 12 of 44 (27%) were internal medicine residents, 9 (20%) were registered or licensed practical nurses, 8 (18%) were primary care attending physicians, and 4 (9%) were pharmacists. Additional details on the breakdown of study participants by clinical site and hospital center can be found in the eTable in Supplement 1.

**Table 1.** Demographic Characteristics of Participants Who Completed Interviews and/or Focus Groups

Characteristic	Participants, No. (%)	
	Patients (n = 69)	Clinicians in primary care or other stakeholders (n = 50)^a^
Sex		
Female	52 (75)	32 (64)
Male	17 (25)	18 (36)
Age, y		
18-24	4 (6)	0
25-34	10 (14)	7 (14)
35-44	5 (7)	6 (12)
45-54	8 (12)	10 (20)
≥55	32 (46)	5 (10)
Not reported	10 (14)	22 (44)
Race and ethnicity		
Asian	8 (12)	11 (22)
Hispanic (any race)	16 (23)	2 (4)
Non-Hispanic Black	9 (13)	5 (10)
Non-Hispanic White	32 (46)	32 (64)
Multiracial or multiethnic	4 (6)	0

^a^ Other stakeholders included a hospital pharmacist, infectious diseases clinician, allergy researcher, pediatrician, health policy expert, and pharmaceutical industry representative.

Several key barriers emerged from the analysis (Table 2). Communication about penicillin allergy emerged as a central roadblock. Clinicians described discomfort initiating these conversations, while patients expressed frustration that the topic was not discussed more often. Time constraints further contributed to clinicians deprioritizing penicillin allergy discussions. Both clinicians and patients reported concerns about the perceived risks of removing penicillin from the allergy list and difficulty accessing an allergy specialist when needed.^35^

**Table 2.** Patient and Clinician Perspectives About Barriers to Penicillin Allergy Delabeling

Barrier	Clinician perspectives	Patient perspectives
Clinicians avoid penicillin allergy conversations as delicate subjects	“For some people who are really convinced about certain things about their history, and objectively, they may be wrong, but it might be what the patient believes. I just want to be careful not to be disrespectful to the patient or do things to damage the relationship.” (Attending physician) “Some patients are a little bit oversensitive if they have many allergies. Sometimes they’ll get extremely anxious.” (Licensed practical nurse)	“Well, if it said you didn’t have an allergy, I would probably say to them, let me tell you again the reasons why I don’t want to take the chance, and say, it’s nice of you to offer that, but the answer is no.”
Patients report never being asked about allergy	Not applicable	“I’ve never really been ever asked about it. They just say you’re allergic and they move on.” “We’re the ones that are experiencing it, so you don’t think we want to talk about it?”
Language and culture related to penicillin allergy	“I wonder if the research team includes someone who is a native Spanish speaker...whether there’s members of the research team who have that linguistic expertise, just to think about because I think cultural too in terms of what is an allergy, and how do you define an allergy.” (Physician)	“Sometimes because there’s a language barrier, it’s not as fair...even when we’re communicating, they will ignore me or neglect me.”
Time constraints	“If you have to have a mammogram every other year, you don’t want to miss the mammogram this year because you didn’t talk about that enough, but you instead talked about penicillin delabeling, so it’s tricky. You have to prioritize because people can’t leave with 8 things to do.” (Attending physician)	Not applicable
Perceived elevated risk of removing a penicillin allergy	“It always feel[s] a little risky to remove it completely...I’m afraid that if I was wrong about them having taken the penicillin medication since then, that might be dangerous for the patient.” (Attending physician)	“No, I don’t want it. I don’t accept it, no. No, because I am also scared. I don’t want to die right now; we’re all going to die but...also—that was a very hard situation on me and my family.”
Limited availability of allergist	“It’s especially hard for us to test because we actually no longer have in-house allergy...We have 1 semiaffiliated allergist, but it’s quite complicated.” (Attending physician)	“When I did look into it, it was months and months out. The appointment.”

Results from the CFIR-ERIC matching tool (Table 3) demonstrated how 5 ERIC strategies translated into 8 penicillin allergy delabeling strategies. The ERIC strategy “create new ways of delivering care” could be operationalized through (1) involving clinicians other than physicians (eg, nurses, pharmacists, and physician assistants), (2) instituting increased access for postpenicillin allergy testing urgent care, and (3) supporting people born in countries that have a primary language other than English in identifying opportunities for penicillin allergy delabeling that make them feel more safe. Identifying clinical champions easily translated into finding primary care champions for penicillin allergy evaluation. One of the physician participants shared their experience as a champion in this respect. The broad ERIC strategy of “using data experts” could more granularly translate to actions through engaging data experts to automate the identification of patients who had safely received penicillin despite an active allergy label and maximizing efficiency through rapid turnaround e-consultation services with allergists. Altering incentive structures, a key ERIC strategy, linked to developing incentives to encourage clinicians in penicillin delabeling, such as making penicillin allergy evaluation a billable service or linking it to pay-for-performance measures. Conducting educational meetings translated to deploying educational meetings for both patients and clinicians, with examples of how patients could be introduced to the concept (“just putting the seed in someone’s head that perhaps they actually don’t have one [a penicillin allergy]” was a quote from a patient) as a first step on the pathway toward future conversations about testing.

**Table 3.** ERIC Strategies to Increase Penicillin Allergy Delabeling With Exemplar Quotes

ERIC strategy applied to penicillin allergy delabeling	Exemplar quote of strategy (speaker)
Create new ways of delivering care	
Involve clinicians broadly in penicillin allergy discussions	“What if you had a nurse or a midlevel or somebody do some of the introductory things? Especially somebody who is really good at interacting with people and listening to concerns and things like that, and reassuring people, and maybe helping them to frame questions and things—as a prelude to going in.” (Nurse practitioner)
Provide after-hours service for patients undergoing delabeling for immediate questions	“Giving people some kind of a beeper or something...so that they can access emergency care immediately...I have kids, and if they’re sick and you don’t have anyone to call, you feel helpless. What, are you going to call the answering service? This idea that even after the hour, or even after the day, you’re not alone.” (Patient)
Support patient-centered strategies for delabeling in people born in other countries	“Because I live alone in the United States, I feel I wouldn’t do the test here. Now I’m wondering maybe if I go back home and I’m around family, then I would be more likely to take a risk and do the test, just because I feel safer around family.” (Patient)
Identify champions	
Find primary care champions	“I have essentially started the initiative here to delabel patients. I think through the lens of wanting to be good antibiotic stewards knowing that a lot of these are not really true allergies, and I think also recognizing through some interesting data that there are just not enough allergists to do this at all. It probably falls to the primary care setting.” (Attending physician)
Use data experts	
Flag patients for clinician review	“There are some informatics approaches that you could take to this...you could check whether this patient has received a penicillin- or cephalosporin-related antibiotic, over time. The next time someone comes through, maybe consider removing the [allergy].” (Resident physician)
Develop asynchronous tools to identify patients for delabeling	“I wonder if for antibiotic prescriptions, where patients with prior antibiotic allergies [are] listed, whether there could be just a [*sic*] allergy e-visit sent to the patient...like, ‘Did you have any drug reaction?’” (Resident physician)
Alter incentive structures	
Reimburse for penicillin allergy evaluation documentation	“If this is something that’s added to the checklist, it needs to be met with some sort of incentive...to truly capture the value that is created by addressing this and preventing the downstream complications of patients falsely listed with a penicillin allergy, that is much more motivating and could actually, perhaps, be another source of very little revenue to primary care.” (Resident physician)
Conduct educational meetings	
Deploy systems to educate patients about penicillin allergy	I just think the concept of actually not having it was never raised, ever. Just putting the seed in someone’s head that perhaps they actually don’t have one.” (Patient)

Abbreviation: ERIC, Expert Recommendations for Implementing Change.

## Discussion

To our knowledge, this was the largest US-based multihospital qualitative study on penicillin allergy evaluation and delabeling as an innovative intervention from the perspectives of patients, clinicians, and other key stakeholders. We found that patients wanted to discuss their penicillin allergy with their clinicians even if some were hesitant about delabeling. In contrast to patients’ interest in discussing their penicillin allergy, clinicians were less comfortable in these conversations. The emergent strategies focused on education, innovative staffing models, harnessing technology, and supporting systems-based interventions to increase confidence and opportunities for primary care clinicians to address penicillin allergy labels.

Several themes emerged as barriers to delabeling that were similar to barriers identified in the UK and Australia, including clinician avoidance of the conversation, time constraints, barriers to accessing allergists, and perceived elevated risk of delabeling to the patient’s health.^36,37,38,39,40^ The continued emergence of themes in our work supports the operationalization of strategies used internationally to support penicillin allergy testing. For example, both the UK and Australia have published implementation strategies focusing on education for nonallergists, with successful increases in penicillin allergy delabeling.^41,42^ Although education has emerged internationally as a key strategy to support the safety of penicillin allergy delabeling, participating in optional training is something that most clinicians do not have time or capacity to do. Education may be 1 component of a successful strategy, but in the US, a multifaceted approach bringing in strategies outside clinician behavior change may be necessary.

An educational strategy deployed in the US would need to address clinician concerns about malpractice. There has been long-term emphasis on collecting an accurate allergy history,^43^ and multiple high-profile legal cases related to antibiotic allergy are in both the lay literature and the research literature.^44^ Most cases resulted in a verdict that showed limited liability from the clinician—a message that has not reached primary care clinicians or has done little to assuage these fears.^45^ Clinicians in our study reported concerns about placing the patient in danger. Educational initiatives would need to address how avoiding penicillins for a patient without confirmed penicillin allergy can lead to negative outcomes.

To date, there has been little attention dedicated to the development of culturally tailored strategies for penicillin allergy delabeling. The literature on improving systems of communication across populations has focused mostly on asthma, atopic dermatitis, and food allergy.^46,47,48^ Perceptions about the safety of health care are intertwined with the community’s views of health care. People who have been negatively impacted by health care may be hesitant to engage in penicillin allergy testing because of their personal beliefs in the validity of the allergy and the potential harm. Many fears are anchored in historical events. For example, batches of compromised penicillins in Cuba have led to mistrust of the antibiotic among people of Cuban heritage, even without an allergy.^49,50^ One way our study adds to the current literature is by specifically examining perspectives of patients and clinicians about how language, race and ethnicity, and insurance impacted penicillin allergy–related conversations. In our study, the importance of having people on the health care team who represent the cultural, racial, and language diversity of the patients emerged.

Financial incentives also emerged as a strategy. Penicillin allergy delabeling is a cost-saving intervention through avoiding expensive antibiotics with harmful adverse effect profiles.^51^ The long-term savings from avoiding antibiotic-resistant infections and adverse effects from antibiotics was not a clear motivator for most clinicians. A congressional bill with bipartisan support, the Penicillin Allergy Verification and Evaluation (PAVE) Act, was introduced in 2024 to incentivize discussions about penicillin with people covered by Medicare.^52^ However, incentivizing clinicians is controversial.^53,54^ Focusing penicillin allergy evaluations on Medicare patients is strategic to target older populations at higher risk of hospitalization and need for antibiotics.^52,55^ Applying these quality metrics can unintentionally punish hospitals that disproportionately care for patients who have public health insurance or medically complex patients.^56^ Routine outpatient care for people who are younger with fewer comorbidities might be a more optimal setting to introduce incentives.

Our work further supports opportunities to use the electronic health record (EHR) to identify people who may not truly have a penicillin allergy. Historically, the EHR has emerged in studies as a barrier to delabeling.^10^ Allergies documented in the EHR are most often recorded before the age of 3 years^57^ and most likely reflect adverse effects, idiosyncratic reactions, or signs and symptoms of viral infections rather than true hypersensitivity.^2^ Penicillin allergies are often repeated incorrectly in EHRs. Even when the penicillin allergy is removed in 1 hospital system, connected electronic records can lead to the allergy being erroneously added back.^58^ Strategies embedded in leveraging data analysts’ expertise emerged in our study, with a specific focus on using the EHR to systematize opportunities for delabeling. All the clinics included in our study used the same EHR system, a common thread that may support future efforts to facilitate delabeling through asynchronous EHR-based messaging to patients and clinicians.

### Limitations

There are several important limitations to our study that should be acknowledged. People who agreed to participate in our study may have had strong feelings about penicillin allergy and increased motivation to support improved systems of penicillin allergy evaluation or health care in general. The clinics we selected as recruitment centers may have had leadership supportive of implementation science research overall, leading the clinicians to be more open to ideas for how clinical practice could change. We were able to translate our interview guide into 3 languages, but resource constraints did not permit further translations to relevant languages for Boston residents, such as Haitian-Creole or Portuguese. Given the number of interviews and focus groups completed across several languages, we used a large research team of 7 people who conducted the interviews and focus groups. While this enhanced the richness of the data captured, it may have also caused variability in the delivery of the interview guide. To ensure consistency, the data were coded by a smaller team using an iterative process. Additionally, the Boston-based research sites represent an urban area, limiting generalizability to rural areas that likely have more restricted access to allergists.

## Conclusions

This qualitative study identified 6 barriers to penicillin allergy delabeling that mapped to 5 implementation strategies for improving equitable access to penicillin allergy evaluation and delabeling. These data may inform future implementation research empirical studies identifying and testing the effectiveness of different delabeling strategies through rigorous evaluation. This study’s findings suggest that the removal of incorrect penicillin allergy labels supports not only the patient’s well-being but also the overall health goals of a high-functioning health care system and optimizing public health.

## Notes

Supplement 1. **eFigure.** Selected Domains/Constructs From Consolidated Framework for Implementation Research 2.0 **eTable.** Number of Participants From Each Clinical Site **eAppendix 1**. Interview Guide for People With Reported Penicillin Allergy **eAppendix 2**. Clinician Interview Guide **eAppendix 3**. Other Stakeholders Interview Guide **eAppendix 4**. Dedoose Codes Export for Project: Aim 2 PENEQ

Supplement 2.
Data Sharing Statement
