# Supplementary material for: Strategies to Increase Equitable Access to Penicillin Allergy Delabeling Interactions: A Qualitative Study
Source: JAMA Netw Open. 2026 Sep 15;9(9):e2633825. doi: 10.1001/jamanetworkopen.2026.33825 (PMC13579434; doi:10.1001/jamanetworkopen.2026.33825)
Supplement: Supplementary file 1 [file jamanetwopen-e2633825-s001.pdf]

Supplementary Online Content

Wurcel A, Crabtree D, Asupoto O, et al. Strategies to increase equitable access to penicillin allergy delabeling interactions: a qualitative study. *JAMA Netw Open*. 2026;9(9):e2633825. doi:10.1001/jamanetworkopen.2026.33825

eFigure. Selected Domains/Constructs From Consolidated Framework for Implementation Research 2.0

eTable. Number of Participants From Each Clinical Site

eAppendix 1. Interview Guide for People With Reported Penicillin Allergy

eAppendix 2. Clinician Interview Guide

eAppendix 3. Other Stakeholders Interview Guide

eAppendix 4. Dedoose Codes Export for Project: Aim 2 PENEQ

This supplementary material has been provided by the authors to give readers additional information about their work.

© 2026 Wurcel A et al. *JAMA Network Open*.

eFigure: Selected Domains/Constructs From Consolidated Framework for Implementation Research 2.0.

This figure highlights the domains and constructs from the Consolidated Framework for Implementation Research updated in 2022[15] which we drew upon when developing our semi-structured interview guide. We addressed a variety of domains in our interview guide: outer setting, implementation process, individuals, inner setting, and innovation. The attention to these domains ensured that we casted a wide net to gain important information from patients, clinicians, and key stakeholders about penicillin allergy evaluation and de-labeling.

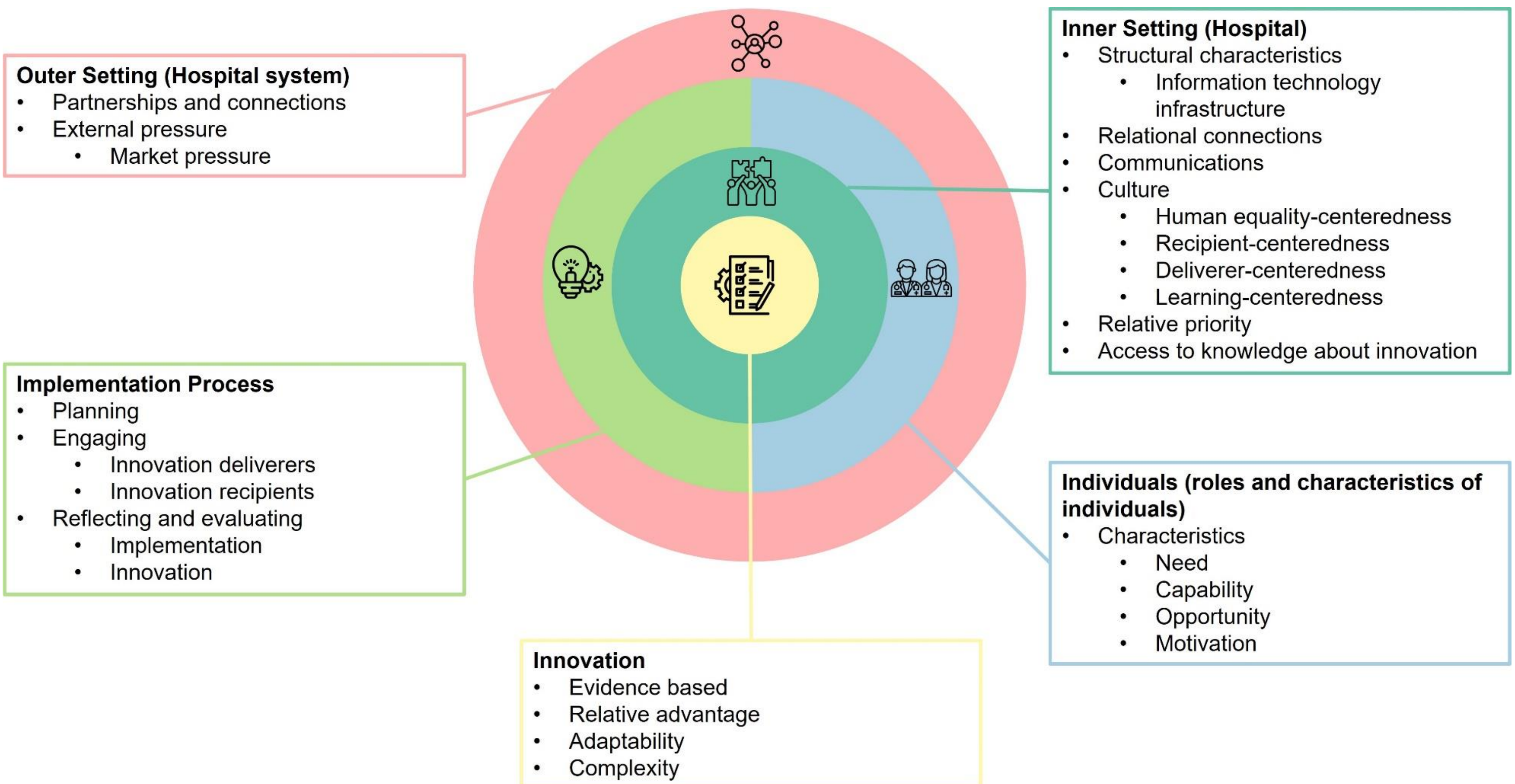

© 2026 Wurcel A et al. *JAMA Network Open*.

eTable: Number of Participants From Each Clinical Site

Hospital	Brigham and Women's Hospital				Massachusetts General Hospital			Tufts Medical Center			Other	Totals
**Clinic**	**Phyllis Jen**	**Longwood**	**Faulkner**	**Other**	**Assembly Row**	**Chelsea**	**Other**	**Lowell**	**Main Campus**	**Other**		
**Patients**	9	16	5	0	9	8	0	8	13	1	0	**69**
**Clinicians**	5	8	0	3	3	5	3	5	10	2	0	**44**
**Stakeholders**	0	0	0	0	0	0	0	0	1	0	5	**6**
**Total from each Clinical Site**	13	24	5	3	12	13	3	13	24	3	5	**119**
**Total from Hospital Center**	**45**				**28**			**40**			**5**	**119**

© 2026 Wurcel A et al. *JAMA Network Open*.

eAppendix 1: Interview Guide for People With Reported Penicillin Allergy

Participant ID _____________

Preferred Language (*circle one*): English Spanish Haitian/Creole Cantonese Other (please list):

If language other than English, communication through (*circle one*):

-Study Staff proficient in language with IRB translated consent form and interview guide

-Interpreter service used.

Interview Date (MM/DD/YYYY) __________

Introduction Script

My name is (INSERT INTERVIEWER NAME). I am a [research assistant/medical student/project manager/fellow] at [Tufts Medical Center/Massachusetts General Hospital]. [If applicable, introduce accompanying study staff members]. Thank you on behalf of my team for agreeing to be part of this interview. We are asking you to participate in this interview because you have an allergy to penicillin, an antibiotic used to treat infections. I would like to ask you questions about your penicillin allergy. There are no “right” or “wrong” answers to these questions.

To make sure that I have an accurate reporting of information from your interview, I will audio record our conversation. If you do not feel comfortable with me audio recording this interview, we can continue the interview without it being recorded. If so, I will be taking notes while I ask the questions. I may ask you to pause or repeat something.

Do I have your permission to audio record this interview? YES NO

Audio recorder turned on (circle): YES NO

If you do not feel comfortable answering a question, it is okay for you to tell me that you do not want to answer. If there’s a question that you would prefer not to answer, you may simply say “pass.” You can also choose to stop this interview at any time and for any reason.

© 2026 Wurcel A et al. *JAMA Network Open*.

Qualitative Interview Guide for People with a Penicillin Allergy

Before we begin, do you have any questions?

Time Interview Started: __________ AM / PM

Time Interview Ended: __________ AM / PM

Interview Questions	Probes
**Section 1: To begin, I will ask you some questions about your history of penicillin allergy:**	
**(1) Tell me how you learned about your penicillin allergy.**	• What were you told about your allergy?<br>• Have you ever taken penicillins like “amoxicillin” or antibiotics like penicillin, cephalexin, and ceftriaxone?<br>• Do you remember what happened when you took penicillin?<br>• Do you think that there is something about your race, ethnicity, or gender that puts you at increased risk for allergy?
**(2)** How do you think having a PCN allergy impacts your chance of having reactions to other medications?	• What are the risks of having a penicillin allergy?<br>• What, if any, are the benefits of having a penicillin allergy?
**(3) On a scale of 1-10, with 1 being the least confident and 10 being the most confident, how confident are you that you are allergic to penicillin?**	• Why did you choose that rating?<br>• What did you base your answer on?
**(4) If you have questions about your penicillin allergy, who would you ask?**	• Have you ever looked up penicillin allergy on the internet? Was it helpful? What did you find?
**(5) Has anyone ever suggested that you may *not* have an allergy to penicillin?**	• How did you respond?<br>• How do you think you would respond?
**(6) Have you heard of penicillin allergy testing?**	• If so, tell me about what you’ve heard?<br>• Who did you talk about it with?

© 2026 Wurcel A et al. *JAMA Network Open*.

Qualitative Interview Guide for People with a Penicillin Allergy

**Section 2: Now I am going to share some information with you:** Research shows that about one ten people in the U.S. population reports a penicillin allergy history, but only 1 in 100 people are truly allergic. Often, people report an allergy to penicillin when they experience a symptom like diarrhea, headache, or fatigue. These are not considered allergies. Sometimes people had a rash when they were children, and the rash was not related to a penicillin allergy; or even if it were, the allergy can get outgrown or go away over time. 8 in 10 people with a confirmed penicillin allergy lose their allergy in a period of 10 years.	
**(7) Have you ever heard of any of this information before?**	• Who/where did you hear it from?
**(8) Now that I told you these penicillin allergy facts, what do you think about your penicillin allergy?**	• How do you think this information may apply to you and your allergy, if at all?<br>• Do you think this research included people like you? People who are your age, gender, race, and ethnicity?
**(9) Have you seen an allergy specialist to discuss if you have a true penicillin allergy?**<br>If YES à CONTINUE BELOW (Q10)<br>If NOà SKIP TO SECTION 3 (Q18)	
**(10)Tell me about the process of scheduling the appointment with the allergy specialist.**	• Were there concerns about the referral process?<br>• Were you able to be served in your preferred language?<br>• How much time did you spend on the phone booking the appointment?<br>• Were you worried about the cost of getting to the appointment or the cost of the office visit?<br>• How did you feel about going for the test when you were originally referred?
**(11)Tell me about the process of seeing the allergy specialist.**	• How many visits did you have for your penicillin allergy evaluation? How much time did you spend at the appointments for your penicillin allergy evaluation?
**(12)Have you had PCN allergy testing?**<br>If YES à CONTINUE BELOW (Q13)<br>If NOà SKIP (Q17)	
**(13)What kind of medical professional did the testing? (e.g., allergist, pharmacist, do not remember)**	• How long did the test take?<br>• What did the testing involve?<br>• Was the test different from what you expected?
**(14)How did the medical professional who tested you describe the results?**	• Do you believe the results of the test?<br>**IF TEST NEGATIVEà Q15**<br>**IF TEST POSITIVEà Q22**
**(15)How did you feel about taking penicillin after finding out that your test was negative for penicillin allergy?**	• After receiving the results that you did not have a penicillin allergy, did a medical professional ever have concerns about giving you penicillin?<br>• Have you taken penicillin or a similar antibiotic since?<br>• How would you feel if prescribed penicillin now?
**(16)When people ask you about your allergies now, what do you say?**	• Do you feel comfortable telling medical professionals that you don't have a penicillin allergy?<br>• Do you feel that medical professionals believe you when you say you don't have an allergy?
**(17)Why did testing not occur?**	• Can you describe the different types of penicillin testing? IF YES à Q22

© 2026 Wurcel A et al. *JAMA Network Open*.

Qualitative Interview Guide for People with a Penicillin Allergy

	IF NOàSection 3
**Section 3: I wanted to take some time to talk about the two types of penicillin allergy tests.** Here is a picture that explains what I will talk about.<br><br>(Hand them the laminated card)<br><br>**One type of test is called the “amoxicillin challenge” or oral challenge.** People with low-risk allergy symptoms (itching, vomiting, nausea, headache, family history of penicillin allergy) often start at this step. Penicillin allergy testing is different than environmental allergy testing. You need to take the drug by mouth without reaction. People take a dose of amoxicillin and then they are watched in the doctor’s office for about an hour. If they do not experience any symptoms, then they do not have a true penicillin allergy.<br><br>**There is another test called the skin test.** People with symptoms such as rash, hives, swelling, or shortness of breath often start testing with the skin test. A skin test is when a small amount of penicillin allergy product is put on the skin. If there is a raised, red area where the penicillin allergy products are, then this means there is an allergy to penicillin. **I**f there is no raised or red area, then the patient will move to the “amoxicillin challenge.”<br><br>Penicillin allergy testing is performed, monitored, and supervised by a healthcare professional. The risk of having a reaction is very small. If you were to develop a skin reaction (hives/itching/redness), you would be given an oral antihistamine to help with the itch or discomfort.	
**(18)How does the information I just gave you make you think about penicillin allergy testing?**	• How is this information different or similar to what you heard about penicillin allergy testing?<br>• Now that you know a little bit more about the penicillin allergy testing, would you be comfortable with the skin testing if you were higher risk?<br>• Would you be comfortable with an amoxicillin challenge if you were lower risk?<br>• Based on the testing options I stated above, which option would you prefer?
**Section 4: There is a shortage of allergy specialists. Many people with penicillin allergy face barriers getting an appointment with an allergist.**	
**(19)If an allergist is not available, who else could do the PCN testing, in your opinion?**	• Your PCP?<br>• A pharmacist?<br>• In the ED?
**(20)If a medical professional did a test that showed you did not have a penicillin allergy, how would that make you feel?**	• How would you feel about taking penicillins if a test showed you that you do not have an allergy?
**(21) After all we have discussed, what do you think about the possibility that you do *not* have a penicillin allergy?**	• Tell me about how you came to that answer.
<u>**Section 4:**</u> **ALL PARTICIPANTS ARE TO BE ASKED THESE QUESTIONS (Q22-29):**	
**(22)What do you think are the risks and benefits of people getting penicillin allergy testing?**	• Would you recommend a penicillin allergy test to someone who thinks they have a penicillin allergy? Why or why not?<br>• What information do you think they should know?
**(23)What could make it easier for patients to discuss penicillin allergy with their doctors?**	

© 2026 Wurcel A et al. *JAMA Network Open*.

Qualitative Interview Guide for People with a Penicillin Allergy

**(24) What do you think about getting an email before your doctor's appointment with information about penicillin allergy and some facts about what is and is not an allergy?**	• What if you received a flyer in the mail?<br>• What if there were information sessions you could attend in your community?<br>• How else could we educate patients about penicillin allergy?
**Section 6:** Thank you for answering these questions. We are going to end with some general questions about your views on healthcare**:**	
**(25) We have heard that people have different levels of trust in their doctor's advice. On a scale of 1-10 (where 1 is low and 10 is high), how much trust do you have in the recommendations you receive from your healthcare team?**	• Tell me about why you chose that number<br>• Who have you encountered in the healthcare system that you trust?<br>• How do you think your trust in healthcare impacts decisions about penicillin allergy testing?
**(26) How do you think your race, gender, or ethnicity impacts your trust in the recommendations you receive from your healthcare team?**	• Have you ever experienced discrimination in the medical system?
I have asked you a lot about penicillin allergy and testing. I want to ask you one more concluding question**: (27)** What are some things that I missed about penicillin allergy that you think are important?	

Participant Self-Identification:

(1.) Self-Identified Race ___________________

(2.) Self-Identified Gender ________________

(3.) Self-Reported Level of Education ________________

(4.) Self-Identified Ethnicity _______________________

(5.) Self-Reported Occupation ____________________

(6.) Self-Reported Age _______

© 2026 Wurcel A et al. *JAMA Network Open*.

eAppendix 2: Clinician Interview Guide

Participant ID ____________

Interview Date (MM/DD/YYYY) _____________

Introduction Script

My name is (INSERT INTERVIEWER NAME). I am a [research assistant/medical student] at [Tufts Medical Center/Massachusetts General Hospital]. [If applicable, introduce accompanying research assistants]. Thank you on behalf of my team for agreeing to be part of this interview. We are asking you to participate in this interview because you may treat patients with a reported allergy to penicillin. I would like to ask you questions about your thoughts about caring for these patients. There are no "right" or "wrong" answers to these questions.

To make sure that I have an accurate report of information from your interview, I will use a digital audio recorder. If you do not feel comfortable with me audio recording this interview, we can continue the interview without it being recorded. If so, I will be taking notes while I ask the questions. I may ask you to pause or repeat something.

Do I have your permission to audio record this interview? YES NO

Audio recorder turned ON (check if yes) ☐

If you do not feel comfortable answering a question, it is okay for you to tell me that you do not want to answer. If there's a question that you would prefer not to answer, you may simply say "pass". You can also choose to stop this interview at any time and for any reason.

Before we begin, do you have any questions?

Time Interview Started: _______ AM / PM

© 2026 Wurcel A et al. *JAMA Network Open*.

Qualitative Interview Guide for Other Stakeholders

Time Interview Ended: _______ AM / PM

Interview Questions	Probes
**<u>Section 1:</u>** To begin, I will ask you some questions about your clinical interactions related to allergies.	
**(1)** Tell me about how you usually identify patients with allergies?	• How do you typically ask patients about their allergies?<br>• Who else in the office asks about allergies?<br>• How do you document allergies in the medical chart?
**(2)** What words do you use when you talk about allergies with patients?	• Do you use words like: sensitivity, hypersensitivity, side effects, intolerances?<br>• What influences your choice of words?
**(3)** How do patients respond when you ask about allergies?	• What medical encounters related to asking patients about allergies stand out?<br>• How has asking about allergies affected your clinical interaction with patients?
**(4)** Can you tell me about your experience caring for patients who have a record of penicillin allergies?	• What gets in the way of caring for people who have penicillin allergies?<br>• When making decisions related to penicillin allergy, what specific resources or factors guide your treatment decision-making?
**(5)** Can you tell me a time you had a discussion with a patient about penicillin allergies?	• What would you have done differently if anything?<br>• How did this experience influence subsequent interactions about penicillin allergy with patients?
**<u>Section 2:</u>** Next, I am going to ask questions about penicillin allergy “de-labeling.” We will use this term to refer to the process of evaluation and subsequent removal of outdated or erroneous entries, sometimes called “de-labeling.” We de-label a patient either after questioning or testing.	

© 2026 Wurcel A et al. *JAMA Network Open*.

Qualitative Interview Guide for Other Stakeholders

**(6)** On a scale of 1-10, how comfortable do you feel talking about penicillin allergy evaluation and de-labeling with your patients?	• Tell me why you chose that number.<br>• Are there certain people you work with who you think feel more or less comfortable with discussing allergies?
**(7)** How important is it to discuss penicillin allergy and de-labeling" with patients?	• What are the benefits of discussing penicillin allergy de-labeling with patients?<br>• What are the risks?
**(8)** **What facilitates the discussion about penicillin allergy de-labelling?**	
**(9)** What are some barriers to discussions about penicillin allergy de-labelling?	
**(10)** I am going to mention a few factors that might play a role in discussions about penicillin allergy. Can you let me know how these affect your discussions? [If already mentioned in 8/9, no not need to mention again]	• Health insurance<br>• Education level<br>• Patient's preferred language<br>• Amount of time in a visit?<br>• Time of the appointment (morning vs. afternoon)?<br>• Supervision of trainee or midlevel?<br>• Patient's health status or co-morbidities?
**(11)** Have you ever removed a penicillin allergy from a patient's record?	• What criteria do you use to decide to remove a penicillin allergy from the patient's record?
**(12)** What barriers are there to changing a penicillin record?	• How easy is it to change a patient's penicillin allergy record?
**(13)** **Are there any resources your clinic could use to help increase penicillin allergy evaluation?**	• What do you think about having pharmacists in the clinic perform penicillin allergy assessments and/or counseling?<br>What about an allergy-trained advanced practice provider or nurse?
<u>Section 3:</u> **Next, I am going to ask you about your knowledge on the process of penicillin allergy testing:**	
**(14)** What is a penicillin allergy evaluation?	
**(15)** Who does the penicillin allergy testing?	• Who can perform the test?<br>• Where does it take place?<br>• How long does it take?<br>• How many visits does it take to confirm or deny penicillin allergy?
**(16)** How do you decide when to refer someone to an allergy service for penicillin allergy testing?	• Why would a patient **not** be referred for penicillin allergy testing?
**(17)** Can you talk about your experiences referring patients for penicillin allergy testing?	• What made you decide to refer the patient?<br>• How did you refer the patient?<br>• What other healthcare professional(s) did you refer the patient to?<br>• How quickly were patients seen by the service?<br>• Were there any barriers to referring to the service?
**(18)** Please tell me about the follow up after the referral?	• How were you, the clinician, informed of the results of the allergy test?<br>• How did you update the patient's records based on the results?<br>• Did you receive a copy of the specialist's note?
**(19)** How does penicillin allergy de-labeling affect patients?	• What are the advantages?<br>• What are the downsides?

© 2026 Wurcel A et al. *JAMA Network Open*.

**(20)** How accessible are allergy specialists to your patients?	• Where is your nearest allergy service?<br>• What do these services provide?<br>• What do you think of these services?
<u>Section 4:</u> **When researchers and clinicians reflect on the race, ethnicity, and gender of patients who are referred for penicillin allergy evaluation and de-labeling, there are variations in the prevalence across different demographic groups. For instance, there are higher numbers of white non-Hispanic/Latino women. In the next section I will ask you some questions about factors that influence if patients are labeled as having a penicillin allergy or referred for penicillin allergy evaluation. Particularly, how the patient's race, ethnicity, or gender influences the likelihood of being labeled or referred for de-labeling.**	
**(21)** Have you observed that there are specific patient groups that are more likely to have a record of penicillin allergies?	• For example, have you noticed any differences in race, ethnicity, or gender of people with penicillin allergy?<br>• Have you noticed specific groups of patients requesting penicillin allergy testing? How does insurance coverage/status affect these requests?
**(22) What patient factors influence your comfort in referring people to an allergy specialist for penicillin allergy delabeling?**	• Are there any medical conditions that impact your decisions?<br>• If a patient has serious mental illness or developmental disability, how does this impact your decision?<br>• What are some ways to improve equitable access to penicillin allergy de-labeling?
Thank you for your time. I'd like to ask you one more concluding question:<br>**(23)** Is there anything else about penicillin allergies or de-labeling you think is important for researchers to know?	

<u>**Participant Self-Identification:**</u>

(1.) Self-Identified Race: ___________________ (4.) Self-Identified Ethnicity: _______________________

(2.) Self-Identified Gender: _________________ (5.) Self-Reported Occupation: _____________________

(3.) Self-Reported Level of Education: _________________ (6.) Self-Reported Age: _______

© 2026 Wurcel A et al. *JAMA Network Open*.

eAppendix 3: Other Stakeholders Interview Guide

Participant ID ____________

Interview Date (MM/DD/YYYY) _____________

Introduction Script

My name is (INSERT INTERVIEWER NAME). I am a [research assistant/medical student] at [Tufts Medical Center/Massachusetts General Hospital]. [If applicable, introduce accompanying research assistants]. Thank you on behalf of my team for agreeing to be part of this interview. We are asking you to participate in this interview because you are someone who works in healthcare and are considered a "stakeholder" in the process of understanding systems of care. I would like to ask you questions about how penicillin allergy impacts healthcare and ways that our team can improve how penicillin allergy is evaluated. There are no "right" or "wrong" answers to these questions.

To make sure that I accurately report what you say, I will use a digital audio recorder. If you do not feel comfortable with me recording this interview, we can continue the interview without it being recorded. If so, I will be taking notes while I ask the questions. I may ask you to pause or repeat something.

Do I have your permission to audio record this interview? YES NO

Audio recorder turned ON (check if yes) ☐

If you do not feel comfortable answering a question, it is okay for you to tell me that you do not want to answer. If there's a question that you would prefer not to answer, you may simply say "pass". You can also choose to stop this interview at any time and for any reason.

Before we begin, do you have any questions?

Time Interview Started: _______ AM / PM

© 2026 Wurcel A et al. *JAMA Network Open*.

Qualitative Interview Guide for Other Stakeholders

Time Interview Ended: _______ AM / PM

<table>
<tr><th>Interview Questions</th><th>Probes</th></tr>
<tr><td>(24) You were identified as someone who is a “stakeholder” in decisions about penicillin allergy, antibiotic stewardship, antibiotic resistance, and/or health equity. Can you describe how your work relates to these topics?</td><td>▪ What are some aspects of your job that relate to penicillin allergy or antimicrobial resistance?
▪ How does your job intersect with health equity?</td></tr>
<tr><td>(25) How common do you think penicillin allergy is?</td><td>▪ If you were to guess, what percentage of people who say they had a penicillin allergy, are truly allergic?</td></tr>
<tr><td colspan="2">I am going to read some facts about penicillin allergy:
About 1 in 10 people in the U.S. population reports a penicillin allergy history, but only 1 in 100 people are truly allergic. Often, people report an allergy to penicillin when they experience a symptom like diarrhea, headache, or fatigue. These are not allergies. Sometimes people had a rash when they were children, and the rash was not related to a penicillin allergy; and even if it were, the allergy can get outgrown or go away over time. 8 in 10 people with a confirmed penicillin allergy lose their allergy in a period of 10 years.</td></tr>
<tr><td>(26) What are your thoughts on this information?</td><td>▪ Are you surprised by this information? Is it consistent with what you know?</td></tr>
<tr><td>(27) What are your thoughts on the public health impact of mislabeled penicillin allergies?</td><td>▪ How important is penicillin allergy mislabeling as a public health issue?</td></tr>
<tr><td>(28) What are some potential negative outcomes for people who have an incorrect penicillin allergy label?</td><td>▪ How might their treatment be affected?
▪ Do antibiotic resistance have broader implications such as psychological effects or healthcare expenses?</td></tr>
<tr><td>(29) Can you share your knowledge about the penicillin allergy evaluation process?</td><td>▪ What are some of the ways that penicillin allergy can be evaluated?
▪ Who can do the evaluation?</td></tr>
<tr><td colspan="2">I am going to read some information about penicillin allergy evaluation and “de-labeling.”
▪ Penicillin allergy “de-labeling” is the process of evaluation and subsequent removal of outdated or erroneous penicillin allergy entries. Patients can be de-labeled after questioning or after questioning and testing. Oftentimes, a label can be removed based on questioning. Testing is not always needed.

▪ Patients with low-risk allergy symptoms (itching, vomiting, nausea, headache, family history of penicillin allergy) can also choose testing. Testing is usually done in an allergist’s office but can be done by other clinicians. Patients take a dose of amoxicillin and then are watched in the doctor’s office for about an hour. If they do not experience any symptoms, then they do not have a true penicillin allergy. People with symptoms such as rash, hives, swelling, or shortness of breath often start with the skin test. If there is a raised or red area where the penicillin allergy products are placed on the skin, this means there is an allergy to penicillin. If there is no raised or red area, then the patient will move to the “amoxicillin challenge” or oral challenge.</td></tr>
</table>

© 2026 Wurcel A et al. *JAMA Network Open*.

Qualitative Interview Guide for Other Stakeholders

**(30)** How does this compare to what you originally thought about penicillin allergy evaluation?	▪ Did you think the process was more/less complicated than this?<br>▪ Do you think people would be interested in this test?
**(31)** What are some barriers that patients might face in penicillin allergy de-labeling?	▪ What are the issues you have seen in your job that could impact access to penicillin allergy evaluation?<br>▪ What issues have seen related to the implementation of penicillin allergy evaluation?
**(32)** What are some barriers that clinicians may face?	▪ How could clinicians overcome these barriers?<br>▪ How can clinicians effectively communicate the difference between true penicillin allergies and other adverse reactions?
**(33)** What are effective ways to tell patients about the risks of penicillin allergy mislabelling?	▪ How can these strategies be integrated into routine clinical practice?
**(34)** What are systems of care that could be refined to improve penicillin allergy de-labeling?	▪ What are some ways that education could be used to improve the current systems?<br>▪ What are some ways to incentivize clinicians to de-label patients?
**(35)** What resources are needed to improve systems of penicillin allergy de-labeling?	▪ How can these resources be integrated into routine clinical practice?
**(36)** Who needs to be involved to improve systems of penicillin allergy de-labeling?	▪ How would these individuals be involved?
**(37)** How do you think healthcare clinicians or leaders could ensure that penicillin evaluation is delivered equitably?	▪ What strategies from examples of equitable healthcare could be applied to improving systems of equitable penicillin evaluation?<br>▪ What outreach strategies could facilitate equity?
**I want to share a recent legislative initiative with you:**<br>The American Academy of Allergy Asthma & Immunology and Congress are currently working together to develop the Penicillin Allergy Verification and Evaluation (PAVE) Act to improve antibiotic stewardship and combat antimicrobial resistance. It mandates routine identification and de-labeling of Medicare patients with penicillin allergy labels in their medical record. This act would require Medicare's initial preventative physical exam and annual wellness visits to include penicillin allergy verification and evaluation.	
**(38)** What do you think of this legislation?	• What challenges do you anticipate clinicians may face when verifying and evaluating a penicillin allergy?<br>• How could these challenges be overcome?<br>• Are there other instances during clinic visits, like the Medicare initial preventative physical, where mandatory penicillin allergy evaluation could be beneficial?
Thank you for your time. I'd like to ask you one last question:<br>**(39)** Is there anything else about penicillin allergies or de-labeling you think is important for the research team to know?	

Participant Self-Identification:

(4.) Self-Identified Race: ____________________ (4.) Self-Identified Ethnicity: ________________________

© 2026 Wurcel A et al. *JAMA Network Open*.

Qualitative Interview Guide for Other Stakeholders

(5.) Self-Identified Gender: ________________ (5.) Self-Reported Occupation: ____________________

(6.) Self-Reported Level of Education: ________________ (6.) Self-Reported Age: _______

© 2026 Wurcel A et al. *JAMA Network Open*.

eAppendix 4: Dedoose Codes Export for Project: Aim 2 PENEQ

Final Codebook from Dedoose Qualitative Data Analysis Software

Allergic to PCN but can take other -cillins Is Weighted: False Description

Allergist appointment suggested to patient Is Weighted: False Description

Allergist did the PCN testing Is Weighted: False Description

Allergist reached out to clinicians about test results Is Weighted: False Description

Allergy specialists' accessibility Is Weighted: False Description

Allergy testing results determine clinician's removal of a PCN allergy record Is Weighted: False Description

Anaphylaxis Is Weighted: False Description

Asking about allergies has negatively affected subsequent clinical interactions Is Weighted: False Description

Asking about allergies has not affected subsequent clinical interactions Is Weighted: False Description

Asthma Is Weighted: False Description

Barrier NOS Is Weighted: False Description

Barriers to care for patients with PCN allergy Is Weighted: False Description

Barriers to changing PCN record in EMR Is Weighted: False Description

Barriers to discussing PCN allergy Is Weighted: False Description

Barriers to PCN allergy testing referral Is Weighted: False Description

Belief in PCN allergy results Is Weighted: False Description

Benefits of discussing PCN allergy Is Weighted: False Description

Benefits of researching PCN allergy Is Weighted: False Description

Can describe different types of PCN tests Is Weighted: False Description

Cannot describe different types of PCN tests Is Weighted: False Description

Childhood experience with PCN allergy Is Weighted: False Description

Clincian feels comfortable discussing alternative PCN medications Is Weighted: False Description

Clincian views on risks of PCN allergy de-labeling Is Weighted: False Description

Clinician asks other people in their office Is Weighted: False Description

Clinician believes it is important to review allergies Is Weighted: False Description

Clinician description of PCN allergy evaluation Is Weighted: False Description

© 2026 Wurcel A et al. *JAMA Network Open*.

Clinician did not discuss PCN allergies during clinical interactions Is Weighted: False Description

Clinician did not follow up with allergist after referral Is Weighted: False Description

Clinician did not receive a copy of allergist note Is Weighted: False Description

Clinician did not update patient's record Is Weighted: False Description

Clinician discusses PCN allergies during clinical interactions Is Weighted: False Description

Clinician does not document allergies Is Weighted: False Description

Clinician has not referred patients for PCN allergy testing Is Weighted: False Description

Clinician has referred patients for PCN evaluation Is Weighted: False Description

Clinician perspective on importance of PCN allergy and delabeling discussion Is Weighted: False Description

Clinician received a copy of allergist note Is Weighted: False Description

Clinician records severity of allergy based on patient report Is Weighted: False Description

Clinician reflections on allergy specialist services Is Weighted: False Description

Clinician report of who does PCN allergy testing Is Weighted: False Description

Clinician shares experience of treating patient with PCN allergy who subsequently has a reaction Is Weighted: False Description

Clinician unsure how to describe a PCN allergy evaluation Is Weighted: False Description

Clinician updated allergies after allergy visit Is Weighted: False Description

Clinicians consult with an allergist Is Weighted: False Description

Clinicians consult with an ID doctor Is Weighted: False Description

Clinicians do not feel comfortable discussing PCN allergy evaluation and de-labeling with patients Is Weighted: False Description

Clinicians document allergies in EPIC Is Weighted: False Description

Clinicians document allergies in Storyboard Is Weighted: False Description

Clinicians feel comfortable discussing PCN allergy evaluation and de-labeling with patients Is Weighted: False Description

Clinicians have not removed PCN allergy from patients' record Is Weighted: False Description

Clinicians have removed PCN allergy from patients' records Is Weighted: False Description

Clinicians knowledge about PCN allergy testing process Is Weighted: False Description

Clinicians use knowledge from medical training Is Weighted: False Description

Clinicians use online resource Is Weighted: False Description

© 2026 Wurcel A et al. *JAMA Network Open*.

Clinicians use patient history to identify allergies Is Weighted: False Description

Clinicians views on advantages of PCN allergy de-labeling Is Weighted: False Description

Clinicians who are comfortable discussing PCN allergies Is Weighted: False Description

Comfortable taking PCN if test showed no PCN allergy Is Weighted: False Description

Comfortable with amoxicillin challenge Is Weighted: False Description

Comfortable with skin test Is Weighted: False Description

Compensation affects PCN allergy discussion Is Weighted: False Description

Competing priorities prevent PCN allergy discussions Is Weighted: False Description

Complex medical history prevents discussion of allergies Is Weighted: False Description

Concerns about cost Is Weighted: False Description

Decreasing mislabeling of kids Is Weighted: False Description

Dentist prescribed the antibiotics Is Weighted: False Description

Description of other stakeholder job Is Weighted: False Description

Disbelief in PCN allergy testing results Is Weighted: False Description

Doctors could ask questions about patient's PCN allergy history Is Weighted: False Description

Doctors should discuss PCN allergy Is Weighted: False Description

Does not remember who did the testing Is Weighted: False Description

Does not think PCN allergy impacts reactions to other medications Is Weighted: False Description

Does not want to see allergist for PCN evaluation Is Weighted: False Description

Education level of patients influences clinicians' word choice Is Weighted: False Description

Embodiment Is Weighted: False DescriptionEmbodiment theory

Emotions if found out no PCN allergy Is Weighted: False Description

Facilitator NOS Is Weighted: False Description

Facilitators to discussion of PCN allergy Is Weighted: False Description

Family helps me with healthcare Is Weighted: False Description

Fear of death or hospitalization from PCN Is Weighted: False Description

Fear of healthcare Is Weighted: False Description

Following Section 3, believes they do not have a PCN allergy Is Weighted: False Description

© 2026 Wurcel A et al. *JAMA Network Open*.

Following Section 3, believes they still do have a PCN allergy Is Weighted: False Description

Genetics and allergy Is Weighted: False Description

Good Quote Is Weighted: False Description

Hard for patients to schedule appointment with allergist due long wait period Is Weighted: False Description

Has heard of PCN allergy testing Is Weighted: False Description

Has not heard of PCN allergy testing Is Weighted: False Description

Has not looked up information about PCN allergy Is Weighted: False Description

Have taken other -cillins since testing Is Weighted: False Description

Health benefit of having PCN allergy Is Weighted: False Description

Health benefit to testing Is Weighted: False Description

Health literacy level of patients influences clinicians' word choice Is Weighted: False Description

Health risk of having a PCN allergy Is Weighted: False Description

Heard about taking PCN in small doses Is Weighted: False Description

Heard information about PCN allergy mislabel rates Is Weighted: False Description

Impact of discrimination Is Weighted: False Description

Impact of health insurance on PCN allergy discussions Is Weighted: False Description

Impact of health literacy/education on PCN allergy discussions Is Weighted: False Description

Impact of health status/comorbities on PCN allergy discussions Is Weighted: False Description

Impact of PCN allergy delabling on patients Is Weighted: False Description

Impact of presence of comorbidities on PCN allergy discussion Is Weighted: False Description

Impact of supervision of trainee or midlevel on PCN allergy Is Weighted: False Description

Impact of time of appointment on discussions about PCN allergy Is Weighted: False Description

Impact of time on PCN allergy discussions Is Weighted: False Description

Information about PCN allergy and de-labeling that's important for researchers to know Is Weighted: False Description

Intolerance is different than allergy Is Weighted: False Description

Learned about allergy from parents Is Weighted: False Description

© 2026 Wurcel A et al. *JAMA Network Open*.

Medical conditions impact PCN allergy de-labeling discussions Is Weighted: False Description

Medical encounters about allergies Is Weighted: False Description

Medical professional told me I was allergic to PCN Is Weighted: False Description

Medical professional told me I was not allergic to PCN Is Weighted: False Description

Memorable encounters about allergies Is Weighted: False Description

Memory of allergic reaction to PCN Is Weighted: False Description

Mental illness or disability does not impact PCN allergy de-labeling discussions Is Weighted: False Description

Mental illness or disability impacts PCN allergy de-labeling discussions Is Weighted: False Description

Mistrust of pharmacists Is Weighted: False Description

Need to be vigilant Is Weighted: False DescriptionPatients report that they are worried about bad things happening in healthcare and express need to always be on the watch

Negative experience referring patient for PCN allergy testing Is Weighted: False Description

Negative views about the idea of flyers before visits Is Weighted: False Description

Negative views on emails before appointments Is Weighted: False Description

Negative views on information sessions Is Weighted: False Description

Never heard information about PCN allergy mislabel rates Is Weighted: False Description

No barriers to changing a PCN record Is Weighted: False Description

No barriers to PCN allergy testing referrals Is Weighted: False Description

No preference for testing options Is Weighted: False Description

No recollection of reaction Is Weighted: False Description

No specific patient groups are more likely to have a PCN allergy record Is Weighted: False Description

Not comfortable with amoxicillin challenge Is Weighted: False Description

Not comfortable with skin test Is Weighted: False Description

Number of visits with allergist Is Weighted: False Description

Other antibiotics available Is Weighted: False DescriptionThis code reflects when someone says that there are lots of other antibiotics available

Other chronic illnesses prevent interest in PCN testing Is Weighted: False Description

Other suggested methods for increasing PCN allergy awareness Is Weighted: False Description

Patient believes no benefits to having PCN allergy Is Weighted: False Description

© 2026 Wurcel A et al. *JAMA Network Open*.

Patient believes PCN allergy limits treatment options Is Weighted: False Description

Patient brings up concern about environmental allergies Is Weighted: False Description

Patient brings up concerns about other drug allergies Is Weighted: False Description

Patient confident (9-10) in PCN allergy Is Weighted: False Description

Patient does not remember the scheduling process Is Weighted: False Description

Patient expresses mistrust of doctors Is Weighted: False Description

Patient has had bad/negative experiences with doctors Is Weighted: False Description

Patient has had good/positive experiences with doctors Is Weighted: False Description

Patient has not seen an allergist Is Weighted: False Description

Patient has seen an allergist Is Weighted: False Description

Patient has trust in doctors Is Weighted: False Description

Patient looked up information about PCN allergy Is Weighted: False Description

Patient not confident (1-2) if they have a PCN allergy Is Weighted: False Description

Patient perspective of on amount of time with allergist Is Weighted: False Description

Patient reflections on ethnicity and allergy Is Weighted: False Description

Patient reflections on gender and allergy Is Weighted: False Description

Patient reflections on race and allergy Is Weighted: False Description

Patient reflections on the internet as a source Is Weighted: False Description

Patient report a negative experience with PCN allergy Is Weighted: False Description

Patient reports no discussion with clinician when reporting allergies Is Weighted: False Description

Patient reports they can/cannot tolerate PCN alternative Is Weighted: False Description

Patient reports to interviewer or clinician about childhood experience with PCN allergy Is Weighted: False Description

Patient reports type of reaction Is Weighted: False Description

Patient somewhat confident (3-8) in PCN allergy Is Weighted: False Description

Patient thinks they do not have a PCN allergy anymore Is Weighted: False Description

Patient thought there was blood work involved in the testing Is Weighted: False Description

Patient told clinician about allergy test results Is Weighted: False Description

Patient unsure about allergy to all -cillins Is Weighted: False Description

Patient wants to do PCN testing Is Weighted: False Description

Patient worried about language barrier Is Weighted: False Description

© 2026 Wurcel A et al. *JAMA Network Open*.

Patient worried about reactions to other medications Is Weighted: False Description

Patient worried about scheduling challenges Is Weighted: False Description

Patient would ask nurse about PCN allergy Is Weighted: False Description

Patient would ask pharmacist about PCN allergy Is Weighted: False Description

Patient would ask their doctor/PCP about PCN allergy Is Weighted: False Description

Patient would not be open to trying PCN testing Is Weighted: False Description

Patient would want more information Is Weighted: False Description

Patient would want more testing before comfortably taking PCNs Is Weighted: False Description

Patient's family is involved in decision-making regarding PCN allergy eval/testing Is Weighted: False Description

Patients brings up concerns about food allergies Is Weighted: False Description

Patients open to PCN testing Is Weighted: False Description

Patients should bring up PCN allergy Is Weighted: False Description

Patients trust themselves more than doctors Is Weighted: False Description

Patients' preferred language influences clinicians' word choice Is Weighted: False Description

Patients' response when asked about allergies Is Weighted: False Description

PCN allergy evaluation is different from stakeholders' thoughts Is Weighted: False Description

PCN allergy evaluation is similar to stakeholders' thoughts Is Weighted: False Description

PCN record can be changed in EPIC allergy section Is Weighted: False Description

PCN record change can be written in notes Is Weighted: False Description

PCN testing sounds similar to other types of medical testing Is Weighted: False Description

Person who told me about my PCN allergy Is Weighted: False Description

Perspectives on allergy-trained advanced practice provider or nurse in PCN allergy assessments Is Weighted: False Description

Pharmacist did the PCN testing Is Weighted: False Description

Places in the chart where PCN allergy documents Is Weighted: False Description

Positive experience referring patient for PCN allergy testing Is Weighted: False Description

Positive views on emails before appointments Is Weighted: False Description

Positive views on flyers before visits Is Weighted: False Description

Positive views on information sessions Is Weighted: False Description

© 2026 Wurcel A et al. *JAMA Network Open*.

Prefers the amoxicillin challenge over the skin test Is Weighted: False Description

Prefers the skin test over the amoxicillin challenge Is Weighted: False Description

Race/gender/ethnicity does not impact trust in healthcare team Is Weighted: False Description

Race/gender/ethnicity negatively impacts trust in healthcare system Is Weighted: False Description

Race/gender/ethnicity positively impacts trust in healthcare system Is Weighted: False Description

Reasons not to refer patient to PCN allergy testing Is Weighted: False Description

Reasons to refer patient with PCN allergy testing Is Weighted: False Description

Reflections of how insurance related to PCN allergy Is Weighted: False Description

Reflections of how race, gender or ethnicity are related to PCN allergy Is Weighted: False Description

Reports PCN allergy to clinicians after having PCN allergy after negative testing Is Weighted: False Description

Research benefit Is Weighted: False Description

Research does not include people like me Is Weighted: False Description

Research includes people like me Is Weighted: False Description

Results of allergy test were updated in EPIC by allergist Is Weighted: False Description

Risk of PCN testing Is Weighted: False Description

Risks of discussing PCN allergy Is Weighted: False Description

Shared decision making Is Weighted: False Description

Side effects of testing Is Weighted: False Description

Someone has not suggested that the patient may not be allergic to PCN Is Weighted: False Description

Someone has suggested they're not allergic to PCN Is Weighted: False Description

Stakeholder awareness of rate of true PCN allergies Is Weighted: False Description

Stakeholder believes their role is connected to health equity Is Weighted: False Description

Stakeholder discusses level of knowledge about PCN allergy evaluation Is Weighted: False Description

Stakeholder does not believe their role is connected to health equity Is Weighted: False Description

Stakeholder mentions financial difficulties for penicillin allergy evaluation and/or testing Is Weighted: False Description

© 2026 Wurcel A et al. *JAMA Network Open*.

Stakeholder reflections of mislabeled PCN allergy and public health Is Weighted: False Description

Stakeholder reflections on clinician barriers Is Weighted: False Description

Stakeholder reflections on clinicians' ability to communicate about PCN allergy Is Weighted: False Description

Stakeholder reflections on examples of equitable healthcare that could be applied to improving system of PCN allergy evaluation Is Weighted: False Description

Stakeholder reflections on how their role relates to PCN allergy/antimicrobial resistance Is Weighted: False Description

Stakeholder reflections on how to integrate resources into clinical practices Is Weighted: False Description

Stakeholder reflections on patient barriers Is Weighted: False Description

Stakeholder reflections on section about rates of true PCN allergy Is Weighted: False Description

Stakeholder reflections on strategies for clinicians to improve communication about allergies Is Weighted: False Description

Stakeholder reflections on strategies for clinicians to integrate PCN allergy mislabeling into clinical practice Is Weighted: False Description

Stakeholder reflections on the broader implications of an incorrect PCN allergy label Is Weighted: False Description

Stakeholder reflections on the impact of an incorrect PCN allergy label on patient care Is Weighted: False Description

Stakeholder reflections on the PAVE Legislation Is Weighted: False Description

Stakeholder reflects on the interest of people with PCN allergy to get testing Is Weighted: False Description

Stakeholder views on who needs to be involved to improve systems of PCN allergy delabeling Is Weighted: False Description

Strategies stakeholders offer to incentivize clinicians to de-label Is Weighted: False Description

Strategies that stakeholders offer to improve education systems Is Weighted: False Description

Strategies to improve equitable access to PCN allergy de-labeling Is Weighted: False Description

Strategy NOS Is Weighted: False Description

Syphilis Is Weighted: False Description

Testing different than expected Is Weighted: False Description

Testing important Is Weighted: False Description

© 2026 Wurcel A et al. *JAMA Network Open*.

Testing safe Is Weighted: False Description

Testing similar to expectations Is Weighted: False Description

Testing unsafe Is Weighted: False Description

Treatment options Is Weighted: False Description

Trust in nurses Is Weighted: False Description

Trust pharmacists Is Weighted: False Description

Tuberculosis Is Weighted: False Description

Uncomfortable taking PCN since test showed no PCN allergy Is Weighted: False Description

Unsure if research includes people like me Is Weighted: False Description

Views about email with PCN allergy facts Is Weighted: False Description

Views about flyer with PCN allergy facts Is Weighted: False Description

Views about information sessions about PCN allergy Is Weighted: False Description

Views on PCN test in the ED Is Weighted: False Description

Views on PCP doing the PCN testing Is Weighted: False Description

Views on Pharmacist doing the PCN testing Is Weighted: False Description

Views on trust in healthcare and PCN allergy testing Is Weighted: False Description

Who asks the patient about allergies Is Weighted: False Description

Words other than allergy that clinicians use to describe reactions Is Weighted: False Description

Would like to learn more about why not allergic to PCN Is Weighted: False Description

Would not recommend PCN testing Is Weighted: False Description

Would recommend PCN testing Is Weighted: False Description

© 2026 Wurcel A et al. *JAMA Network Open*.
