# Supplementary material for: Strategies to Increase Equitable Access to Penicillin Allergy Delabeling Interactions: A Qualitative Study
Source: JAMA Netw Open. 2026 Sep 15;9(9):e2633825. doi: 10.1001/jamanetworkopen.2026.33825 (PMC13579434; doi:10.1001/jamanetworkopen.2026.33825)
Supplement: Supplementary file 2 [file jamanetwopen-e2633825-s002.pdf]

Data Sharing Statement

Wurcel. Strategies to Increase Equitable Access to Penicillin Allergy Delabeling Interactions. *JAMA Netw Open*. Published September 15, 2026. doi:10.1001/jamanetworkopen.2026.33825

Data
Data available: No
